# Clinical Implementation of Cone Beam Computed Tomography-Guided Online Adaptive Radiation Therapy in Whole Breast Irradiation

**DOI:** 10.1016/j.adro.2024.101664

**Published:** 2024-11-05

**Authors:** Koen J. Nelissen, Wilko F.A.R. Verbakel, Judith G. Middelburg–van Rijn, Barbara L.T. Rijksen, Marjan A. Admiraal, Jorrit Visser, Jessica van der Himst, Karin N. Goudschaal, Ewa Bucko, Ben J. Slotman, Angelique R.W. van Vlaenderen, Desiree H.J.G. van den Bongard, BREAST-ART study group

**Affiliations:** a Department of Radiation Oncology, Amsterdam UMC location Vrije Universiteit Amsterdam, Amsterdam, The Netherlands; b Cancer Center Amsterdam, Cancer Treatment and Quality of Life, Amsterdam, The Netherlands; c Varian Medical Systems, Radiotherapy Solutions, Palo Alto, California; d Cancer Center Amsterdam, Cancer Biology and Immunology, Amsterdam, The Netherlands

## Abstract

**Purpose:**

In postoperative breast irradiation, changes in the breast contour and arm positioning can result in patient positioning errors and offline replanning. This can lead to increased treatment burden and strain on departmental logistics because of the need for additional cone beam computed tomography (CBCT) images or even a new radiation therapy treatment plan (TP). Online daily adaptive radiation therapy (oART) could provide a solution to these challenges. We have clinically implemented and evaluated the feasibility of oART for whole breast irradiation.

**Methods and Materials:**

Twenty patients treated with postoperative whole breast right irradiation (5 × 5.2 Gy) were included in BREAST-ART, a prospective single-arm trial. The dosimetry of the reference TP calculated on the daily anatomy and adaptive TP were compared. Duration of the oART workflow, in-house satisfaction questionnaires, and acute toxicity (National Cancer Institute Common Terminology Criteria for Adverse Event v5.0) were collected. The oART workflow was evaluated by investigating the impact of manual corrections of influencer and target contours on treatment time and quality.

**Results:**

In the first 17 patients (85 fractions), the on-couch time, ie, the time between the end of CBCT1 and CBCT3, was a median of 13.8 minutes (range, 11–25). Retrospective evaluation of the use of the influencer (ie, breast) in 4 patients (20 fractions) and manual correction of the most cranial and caudal target contours (ie, 4 mm) in 10 patients (36 fractions) was done. This resulted in a reduced on-couch time in the last 3 clinical patients to a median of 13.0 minutes (range, 11–19). No grade 3 or higher toxicity was observed, and 19 of 20 patients indicated that they preferred the same treatment again. Skin marks for patient positioning during treatment were no longer necessary.

**Conclusions:**

This study showed the feasibility, challenges, and practical solutions for the implementation of oART for breast cancer patients. Future work will focus on more complex breast indications, such as whole breast, including axillary nodes, to further investigate the benefits and challenges of oART in breast cancer.

## Introduction

In most women with early-stage breast cancer, breast-conserving surgery is followed by postoperative irradiation to reduce the risk of locoregional recurrence and to improve disease-free survival.^1^ Standard image guided radiation therapy (IGRT) workflows for breast cancer patients include on-couch positioning using laser lines (or surface-guided if available), registration of cone beam computed tomography (CBCT), and radiation therapy (RT) planning computed tomography (CT) images, followed by a couch shift. Correct positioning of the target based on skin marks can be challenging. Breast tissue is nonrigid, influenced by the exact positioning of the arm above the head, and can still vary regardless of positioning efforts.^2^^,^^3^ A study by Hattel et al^4^ investigated positioning uncertainty using the residual root mean square between setup with skin marks and planning CT-CBCT image matching and correction, which was 5.4 mm. Another study by Stanley et al^5^ evaluated the shift vector between setup with skin marks and planning CT-CBCT matching, which was, on average, 14 mm (SD, 0.7). In case the breast contour cannot be matched between the CBCT and the planning CT images within geometric tolerance, repositioning of the breast, the arm, or the entire patient is necessary, followed by another setup CBCT. This positioning and acquisition of additional CBCTs can result in a prolonged treatment time on the linear accelerator. This can increase the treatment burden for the patient and the need for health care resources, such as a new RT planning CT images and RT plan. In some cases, repositioning does not suffice, and offline replanning is needed because of breast contour changes during RT treatment, such as variations in seroma or breast edema.^6^

Since 2010, conventional dose fractionation regimens, consisting of 50 Gy in 25 fractions, were replaced by hypofractionated schedules (ie, 42.56 Gy in 16 fractions, 40.05 Gy in 15 fractions) with equal oncological outcomes and similar or less toxicity.^7^, ^8^, ^9^ Since 2020, ultrahypofractionated schedules have been implemented in patients treated with local RT based on the results of the "FAST" (5 × 5.7 Gy) and "FAST-Forward" trials (5 × 5.2 Gy).^9^^,^^10^ Especially for the FAST-Forward ultrahypofractionated RT scheme, offline replanning can be challenging as there are only 5 treatment fractions in 1 week. At our department, offline replanning occurs in 9% to 15% of breast RT cases (Methods E1). This can cause high pressure on the logistics of the RT department to perform new RT planning CT, contouring, and treatment planning before the next fraction 1 day later to prevent increased overall treatment time.

Online adaptive RT (oART) allows adaptation of the treatment plan during each RT fraction according to the anatomy and patient position of the day. A recent study showed the feasibility of breast oART with digital treatment simulations, reporting good target propagation and target coverage.^11^ Another study showed the feasibility of oART in stereotactic partial breast irradiation, achieving improved target coverage, similar organ at risk (OAR) goals, and reduced planning target volume (PTV) margins compared with IGRT treatment. oART may improve the OARs sparing by reducing PTV margins used for day-to-day variation in setup uncertainty.^12^^,^^13^ Consequently, oART can reduce the treatment burden for the patient and logistic challenges at the RT department compared with offline replanning. Since there is no need for an extra planning CT, no additional waiting times, or unexpected treatment interruptions for the patient, neither offline delineation of new contours nor treatment planning in case of positional or anatomic changes is necessary. On the contrary, oART workflows could require longer treatment timeslots and the presence of a radiation oncologist and/or medical physics expert at the machine. We report the feasibility and clinical implementation of oART for right-sided whole breast irradiation after breast-conserving surgery.

## Methods and Materials

### Study design

The breast adaptive radiotherapy (BREAST-ART) trial (NCT05727553) is a prospective cohort study that was initiated in June 2022. The Amsterdam UMC ethics committee determined that this study is not subject to the Dutch law for Medical Research on Human Subjects (IRB 2021.0624). The primary endpoint is time spent on the oART workflow. The secondary endpoints are patient experience, dosimetric data, and treatment-associated toxicity.

Eligible patients in this first cohort were identified based on (1) indication for postoperative right-sided whole breast irradiation (WBI). Treatment was ultrahypofractionated, delivering 26 Gy in 5 fractions of 5.2 Gy on consecutive workdays and (2) curative intent. For the initial implementation of oART in breast cancer, the easiest site (WBI right) was chosen. Also, at the start of the BREAST-ART trial, the breath-hold technique for the adaptive workflow was not available yet in our clinic, leading to the exclusion of left-sided breast targets. This patient group was included in the later stages of the study. Other patients with more complex RT breast indications, including axillary lymph nodes, partial breast irradiation, and tumor bed boost, were also included in the later stages of the study.

### Treatment planning

Clinical target volume (CTV) was defined as the whole breast in all patients. PTV was constructed by expanding the CTV by 5 mm in all directions. All targets were cropped to 5 mm below the body contour before plan optimization. A ring structure was created around the PTV up to 30 mm (ie, ring structure) and used to suppress hotspots outside the PTV. OARs included the contralateral breast, heart, and lungs. The standard RT planning technique for WBI used at our department was hybrid intensity modulated RT (IMRT) with tangential beam setup.^14^ However, in the current version of the used CBCT-guided adaptive treatment system “Ethos” (Varian, Siemens Healthineers), hybrid treatment planning is not possible. Therefore, a 4-tangential beam IMRT technique was used, and the tangential beam angles were optimized automatically using an in-house developed script in Eclipse (Methods E2).^15^ The beam setup was imported into the Ethos treatment planning system (TPS) and used for automated treatment planning based on planning templates, resulting in the reference treatment plan (TP_R_). Templates were developed and tested using a replica of the clinically used oART system provided by the manufacturer (data not shown). Treatment planning templates were developed based on clinical dose volume histogram criteria for the OARs and targets and tested in the Ethos TPS (Table 1). In addition to the goals shown in Table 1, 3 goals were added to the template: ring structure minimum dose in 1cm3 (D0.1 cm3) ≤ 27.82 Gy (107%), D0.1 cm3 ≤ 28.60 Gy (110%), and lungs mean dose (Dmean) ≤ 3 Gy. The template goals were incidentally changed when a clinical goal was not satisfied.

**Table 1:** Clinical goals per fraction are shown for the clinical target volume cropped 5 mm from the body contour (CTV-05), planning target volume cropped 5 mm from the body contour (PTV-05) , right lung, left breast, and heart. *Abbreviations:* DXX = minimum dose delivered to XX of the volume, Dmean = mean dose in the volume, VX Gy = percentage of volume recieving atleast X Gy.

Target	CTV-05 D98% (%,)	PTV-05 D98% (%,)	PTV-05 D2% (%)	PTV-05 D0.1 cm3 (%)	PTV-05 Dmean (%)
Clinical goal	≥95 (not in template)	≥95	≤107	≤112 (≤aim 107%)	≥99 and ≤101%
TP_R_	96.7 (96.2-96.9)	95.6 (95.0—95.9)	103.7 (103.2—104.3)	107.5 (105.5—112.9)	100.1 (99.2—100.6)
TP_S_	96.2* (89.6—97.5)	93.8* (76.8—96.2)	104.0* (103.1—107.5)	108.1* (105.2—113.8)	99.7* (97.7—101.0)
TP_A_	96.7* (96.2—97.5)	95.7* (95.1—96.0)	103.7* (102.9—104.7)	107.1* (105.5—115.6)	100.1* (99.0—100.7)
**OARs**	**Lung right V5 Gy (%)**	**Lung right V20 Gy (%)**	**Breast left Dmean (cGy)**	**Heart Dmean (cGy)**	**Lungs Dmean (cGy)**
Clinical goal	≤20	≤10	≤20	≤16	≤60
TP_R_	18 (9—27)	4 (1—10)	3 (2—6)	7 (6—14)	37 (22—69)
TP_S_	17 (7—27)	4 (0.2—18)	3 (2—6)	7 (5—9)	37 (21—69)
TP_A_	17 (8—29)	4 (0.3—11)	3 (2—8)	7 (5—14)	37 (25—72)

Median dose (range) is shown for TP_R_ (pretreatment), TP_S_ (pretreatment plan on daily anatomy), and TP_A_ (new treatment plan on daily anatomy). The dose from the 3 cone beam computed tomographies was not included in the presented data.

*Abbreviations:* CTV = clinical target volume; Gy = gray (J/kg); OAR = organ at risk; PTV = planning target volume; TP_A_ = adaptive treatment plan; TP_R_ = reference treatment plan; TP_S_ = scheduled treatment plan.

⁎ Significant difference (*P* < .05) between TP_S_ and TP_A_.

Normally, in Eclipse breast treatment planning, the final fluence map of each beam is expanded manually in the medial-lateral direction as a skin flash technique to improve treatment robustness for intrafraction motion. Unfortunately, this technique is not possible in the Ethos TPS. A technique was developed specifically for Ethos TPS, which forces the optimizer to produce a similar fluence map by using a digital “mini-bolus” (Methods E3).

### Treatment procedure

**Figure 1:**
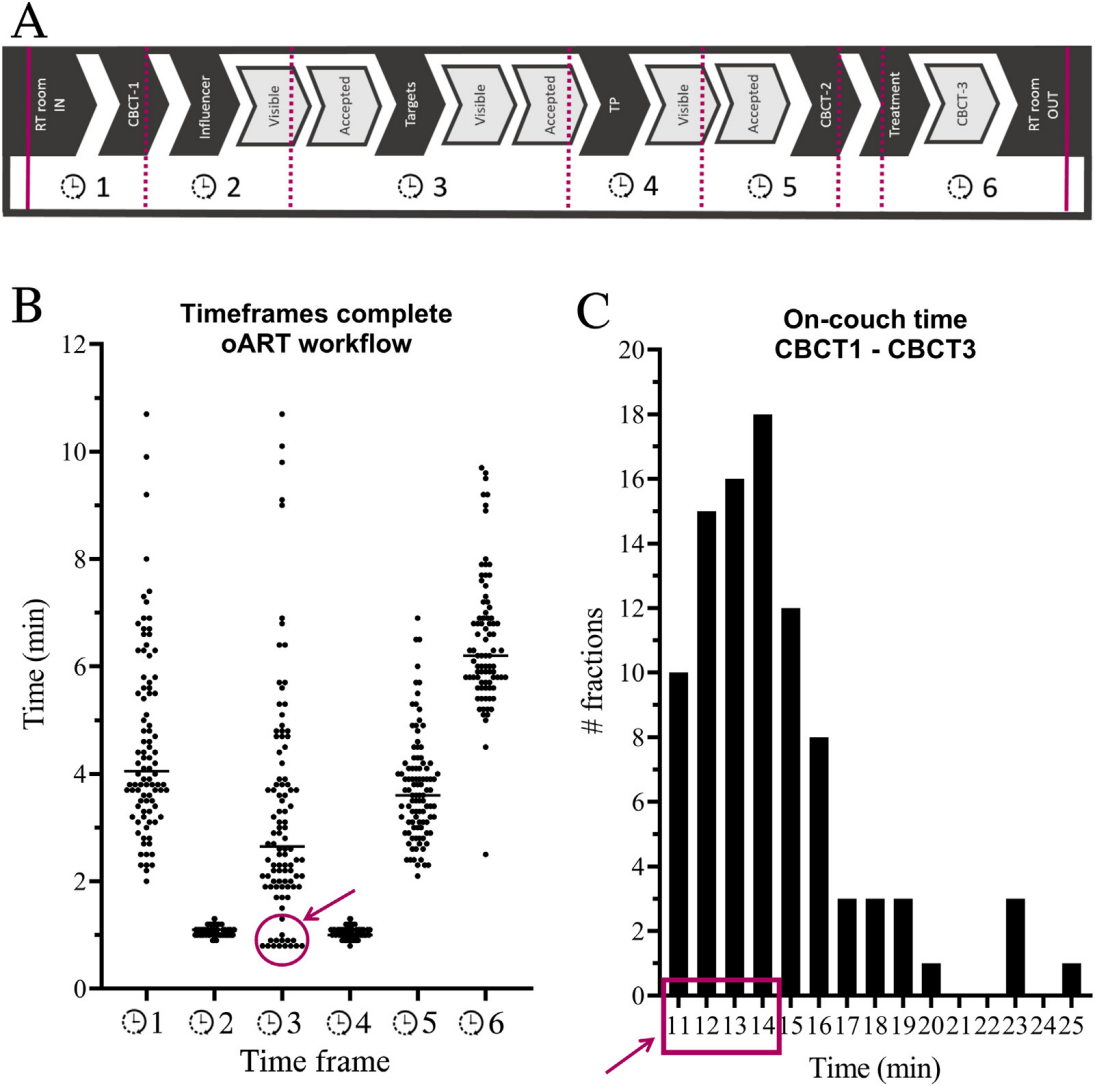
Overview of the duration of the online adaptive radiation therapy (oART) procedure. (A) The workflow and corresponding timeframes. (B) An overview of each time frame per fraction: time frame 1 (enter + cone beam computed tomography [CBCT]1) represents 93 fractions, time frame 6 represents 82 fractions, and other time frames, 100 fractions. The circle and arrow indicate 15 treatment fractions without influencer structures. (C) A histogram of the treatment time between CBCT1 and CBCT3, with a total of 93 fractions. The rectangle and arrow include all 15 treatment fractions without influencers. *Abbreviations:* RT = radiation therapy; TP = treatment plan.

The on-couch workflow during each RT fraction is shown in Fig. 1A,^16^, ^17^ including the definition of time frames (TFs). TF1 includes patient arrival in the treatment room, procedure explanation, patient setup based on skin marks or anatomic landmarks, and the first CBCT. During TF2, the system generates a synthetic CT (sCT) using a deformable image registration of the planning CT (pCT) to the CBCT; this sCT contains the correct density information from the pCT deformed to the daily anatomy. The sCT is used for plan optimization and dose calculation during the online workflow. At the end of TF2, the ipsilateral breast is generated on the sCT (representing daily anatomy). The ipsilateral breast is used to guide the target deformation and is defined as an influencer structure. The influencer structure is checked and manually corrected by the radiation technologist (RTT) (specifically trained in the oART workflow) supervised by a dedicated breast radiation oncologist. In TP3, target contours were propagated by an influencer-guided deformable image registration. Target contours were checked and corrected, if necessary, by the RTT supervised by a dedicated breast radiation oncologist. During TP4, an automatic optimization of the adapted treatment plan (TP_A_) based on the planning templates was performed on the sCT. Additionally, the dose was calculated on the sCT using the original TP_R_, which is defined as the scheduled treatment plan (TP_S_). Next (TP5), the RTTs, radiation oncologist, and medical physics expert chose between TP_A_ and TP_S_ for delivery, followed by verification of the dose distribution and dosimetric parameters. This was followed by a second CBCT2 for position verification and couch correction if necessary. In the final part of the workflow (TP6), treatment delivery was performed, followed by a third CBCT to check for the intrafraction motion during treatment delivery. The dose for CBCT1, using the thorax protocol, was approximately 70 mGy per scan, and for CBCT2 and CBCT3, using the breast protocol, it was approximately 14 mGy (internal data, measured based on guidelines by the Dutch committee for dosimetry “NCS,” report 32). The CBCT dose was not considered during treatment planning. Standard departmental quality assurance was done for the TP_R_ and TP_A_ using Mobius software (Varian, Siemens Healthineers) to perform a secondary dose check.

### Evaluation of the adaptive workflow

Treatment time data (Fig. 1A) on entering and exiting the treatment room were recorded by RTTs, and additional time data were extracted from Digital Imaging and Communications in Medicine (DICOM) data (exported from Ethos TPS) using a MATLAB (version R2021a) script. Dosimetric data for clinical goal dose volume histogram points (Table 1) and target volumes were collected for the TP_R_, TP_A_, and TP_S_. The Wilcoxon signed rank test (paired) was used to compare dosimetric data between the TP_R_, TP_A_, and TP_S_.

The impact of manual corrections of the influencer or CTV on the treatment plan was evaluated by replicating the workflow without any corrections of influencers or target contours for the first 10 patients in a testing environment identical to the clinical software. The aim of this evaluation was to evaluate the effect of using the propagated target contours without any corrections on the optimized treatment plans to assess whether the manual corrections of the propagated target contours were clinically relevant. This resulted in emulated adaptive treatment plans (TP_AE_) optimized for propagated target contours without manual correction. The clinical goals for CTV and OARs were evaluated for TP_AE_ and TP_A_ on both clinically used manually corrected and automatically corrected structures. This evaluation was done for all fractions of the first 10 patients in which manual corrections were performed during the clinical workflow.

Additionally, the impact of an influencer structure was evaluated on the quality of target propagation. For 4 patients, the workflow was replicated for all 20 fractions in the testing environment without the use of an influencer structure. The resulting target structures were evaluated qualitatively on the CBCT by a radiation oncologist in comparison with the original reference planning structures. The radiation oncologist assigned the following definitions per fraction: no edits, minor edits (<10% of slices), and major edits (>10% of slices).^18^^,^^19^ These were the edits needed for the target structures created by the oART system to match clinical standards.

In-house developed patient questionnaires were used to measure patient experience and satisfaction during the on-couch workflow (Methods E4). Questionnaire answer options were based on a 4-point Likert scale, in which patients had to score the level of agreement with 10 different statements. This brief questionnaire was filled in after the first and fifth RT fractions. The last 5 patients filled in only 1 questionnaire after the fifth RT fraction. Additionally, radiation-associated toxicity was collected using the Common Toxicity Criteria Adverse Events version 5.0^20^ at baseline (ie, postoperatively and before the start of RT) and 1 month and 3 months after the end of treatment. Questionnaires and toxicity were evaluated using descriptive statistics.

## Results

### Study data set

Between June 2022 and September 2023, 20 patients were included in the BREAST-ART trial (NCT05727553) for postoperative WBI (ie, 26 Gy/5 fractions). All patients were female and presented with an invasive carcinoma (n = 17) or ductal carcinoma in situ (n = 3) of the breast (Table 2). During all fractions, TP_A_ was chosen and used for treatment delivery. Fourteen patients were treated at the main Department of Radiation Oncology of the Amsterdam UMC and 6 at our satellite locations at the Flevoziekenhuis (Almere, The Netherlands) by another team of oART-trained RTTs. Planning CT simulation was performed, including semipermanent line skin marks for 13 patients. All subsequent patients were treated without any skin marks or surface-guided RT (SGRT). Three patients were planned using the mini-bolus technique.

**Table 2:** Patient and tumor characteristics

No. of patients	20
Median age (range), y	59 (3384)
Tumor location	-
Lateral upper quadrant, n	12
Lateral lower quadrant, n	5
Medial lower quadrant, n	3
Target volume (CTV-05) on planning CT (range), cm3	782 (3112113)

All patients had right-sided breast cancer treated with breast-conserving surgery and postoperative whole breast irradiation (26 Gy/5 fractions).

*Abbreviations:* CT = computed tomography; CTV-05 = clinical target volume cropped 5 mm from body contour.

### Treatment procedure data

**Figure 2:**
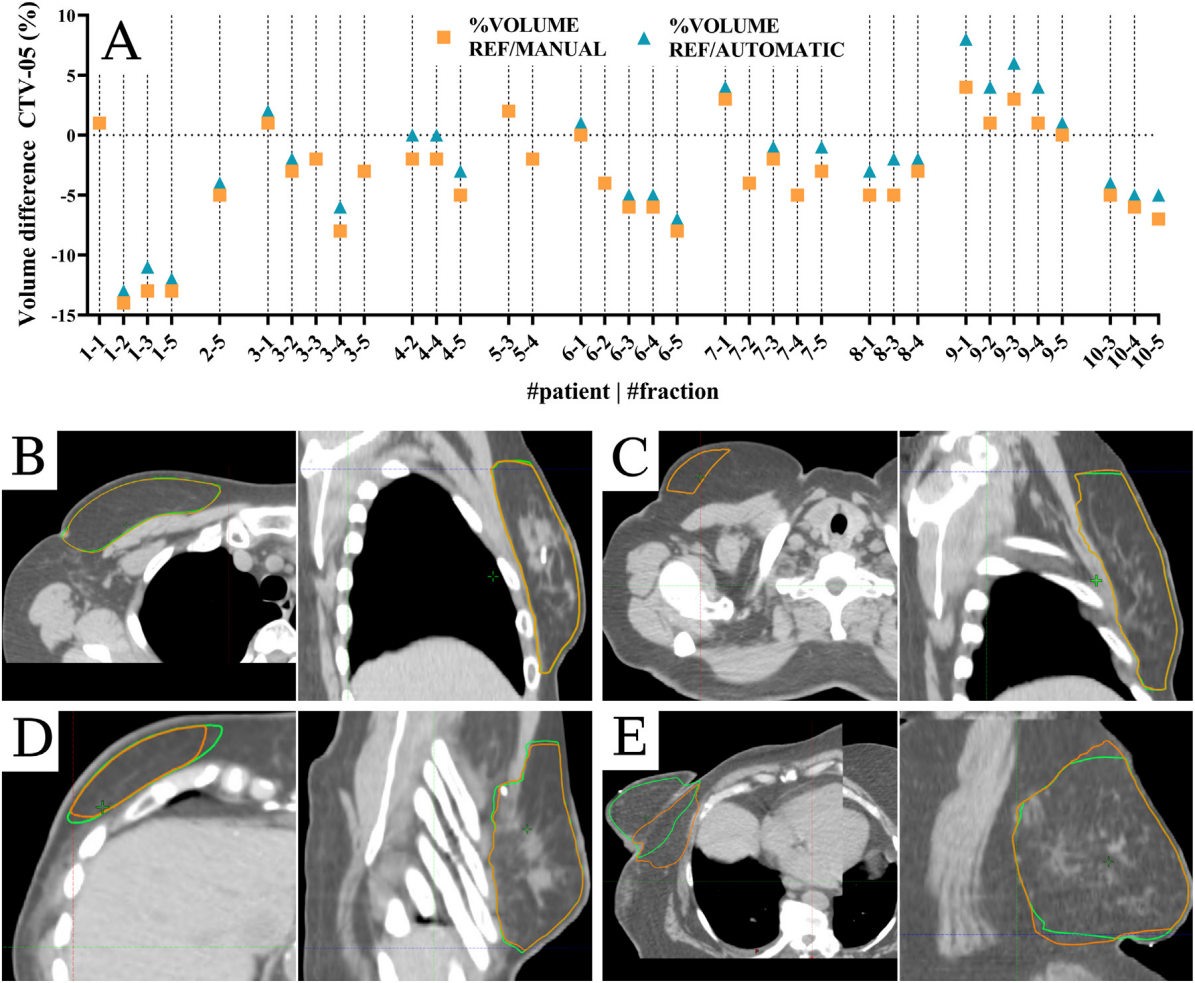
(A) Volume differences between the reference (Ref) volume divided by manually corrected volume and automatically corrected volume. (B-E) Synthetic computed tomography: orange = automatically corrected contours, and green = manually corrected contours. (B) Axial and sagittal view of patient 2 fraction 5, (C) axial and sagittal view of patient 3 fraction 2, (D) axial and sagittal view of patient 8 fraction 1, and (E) axial and sagittal view of patient 9 fraction 5. *Abbreviations:* CTV-05 = clinical target volume cropped 5 mm from the body contour.

All patients completed all prescribed fractions successfully. The duration of the oART procedure (Fig. 1A) was collected in 82 of 100 fractions; the other fractions had partially missing data on the times that the patient entered and left the treatment. The median (range) oART procedure duration was 20.0 minutes (14–35) (n = 82 fractions), including entering and exiting the treatment room by the patient (Fig. 1). Data on the end of treatment were partially missing because of errors in DICOM data; 93 of 100 fractions were collected. The median (range) on-couch duration (ie, start CBCT1 until end CBCT3) was 13.8 minutes (10.7–24.6) (n = 93 fractions). In total, 85 of 100 fractions used influencer structures, which is elaborated on in the next section. The RTT, under the supervision of the breast radiation oncologist, manually corrected the influencer structures during 46 of 85 fractions and the propagated target contours during 14 of 100 fractions. Five fractions with an on-couch time of more than 20 minutes needed a longer target/influencer manual correction time (range: 9–11 minutes). In patient 9, 4 fractions required 6 to 11 minutes of manual contouring because of a discrepancy between the planned contour and the (smaller, Fig. 2A) desired anatomy of the day contour combined with bad deformation by the system.

The median number of Monitor Units was 1478 (range: 1172–2125) and 1526 (range: 1200–2049) in TP_A_ and TP_R_, respectively. Couch corrections just before a treatment based on matching between CBCT1 and CBCT2 were, on average, 0.13 cm (SD, 0.15), −0.01 cm (SD, 0.16), and 0.01 cm (SD, 0.12) for vertical, longitudinal, and lateral directions, respectively. The dosimetric results and clinical goals are shown in Table 1 for TP_A_ and TP_S_.

### Evaluation of the adaptive workflow

The use of the influencer was evaluated in 4 patients (20 fractions). The target contours propagated with the addition of the influencer structures resulted in 10 minor edits (<10% slices), 1 major (>10% slices), and 14 with no edits. Targets propagated without the influencer structures resulted in 6 minor edits, 0 major, and 19 with no edits. Based on the results of this evaluation, using the breast as an influencer structure was considered to have no added value. The last 3 patients were treated without using influencer structures. Using no influencer in clinical patients resulted in a shorter time between CBCT1 and CBCT3 for all 15 fractions; this time was <15 minutes (Fig. 1).

To evaluate the influence of manual target contour correction in oART workflow, we evaluated 36 fractions of patients 1 to 10, for which manual corrections of the target contours and/or influencer were performed. These fractions were emulated to evaluate the clinical relevance of manual target contour correction. Differences in volumes between manually corrected and automatically corrected contours are shown in Fig. 2. Two typical examples of contour differences between manual and automatic corrections are shown in Fig. 2B, C. Figure 2D, E show nontypical examples of contour differences. In most fractions, the case of Fig. 2C was observed, where slight differences in volumes were because of differences in contours on the most cranial and caudal slices of the target.

**Figure 3:**
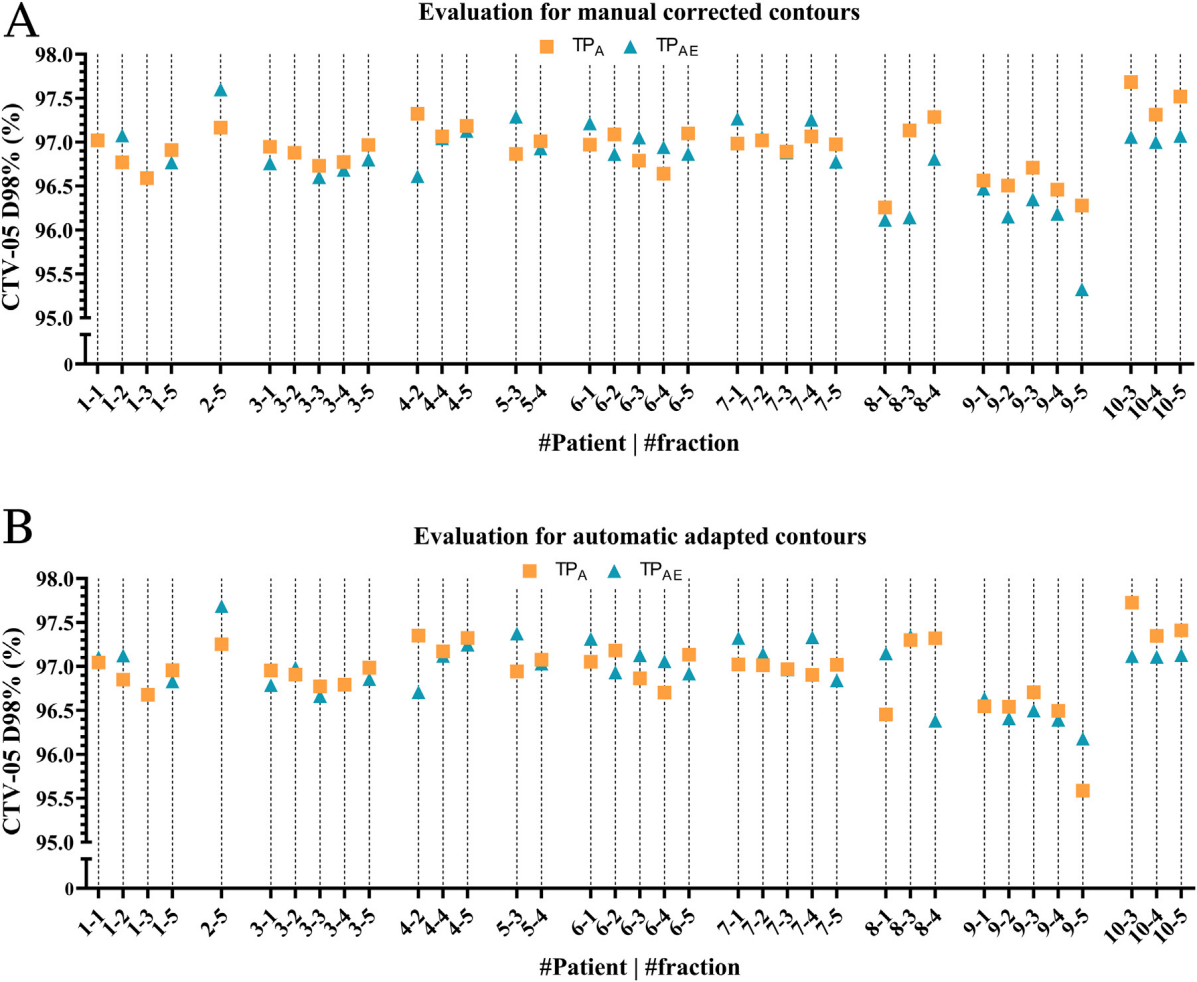
Plan optimization differences for manual corrected (A) and automatic corrected contours (B). *Abbreviations:* CTV-05 D98% = minimum dose delivered to 98% of the clinical target volume cropped 5 mm from the body contour; TP_A_ = treatment plan optimized on manually corrected contours; TP_AE_ = treatment plan optimized on automatically corrected contours.

Figure 3 shows an overview of the differences in target coverage for all 36 fractions. Additionally, other clinical goals for PTV and OARs were evaluated by comparing the median (range) dose difference between TP_A_ and TP_AE_ on the manually corrected contours. No significant differences were observed. Based on the results regarding contouring and target coverage (Figure 2, Figure 3), we decided not to correct contours less or equal to the 2 most cranial or caudal slices of the target volume, expediting the oART workflow further by reducing the contouring time during treatment.

In general, patients were satisfied with the treatment procedure. All patients except for 1 indicated to prefer the same type of treatment again. Another patient experienced anxiety during the treatment procedure with an unknown cause. Three patients reported dissatisfaction with communication while on-couch. Results per question asked are included in Methods E4.

**Figure 4:**
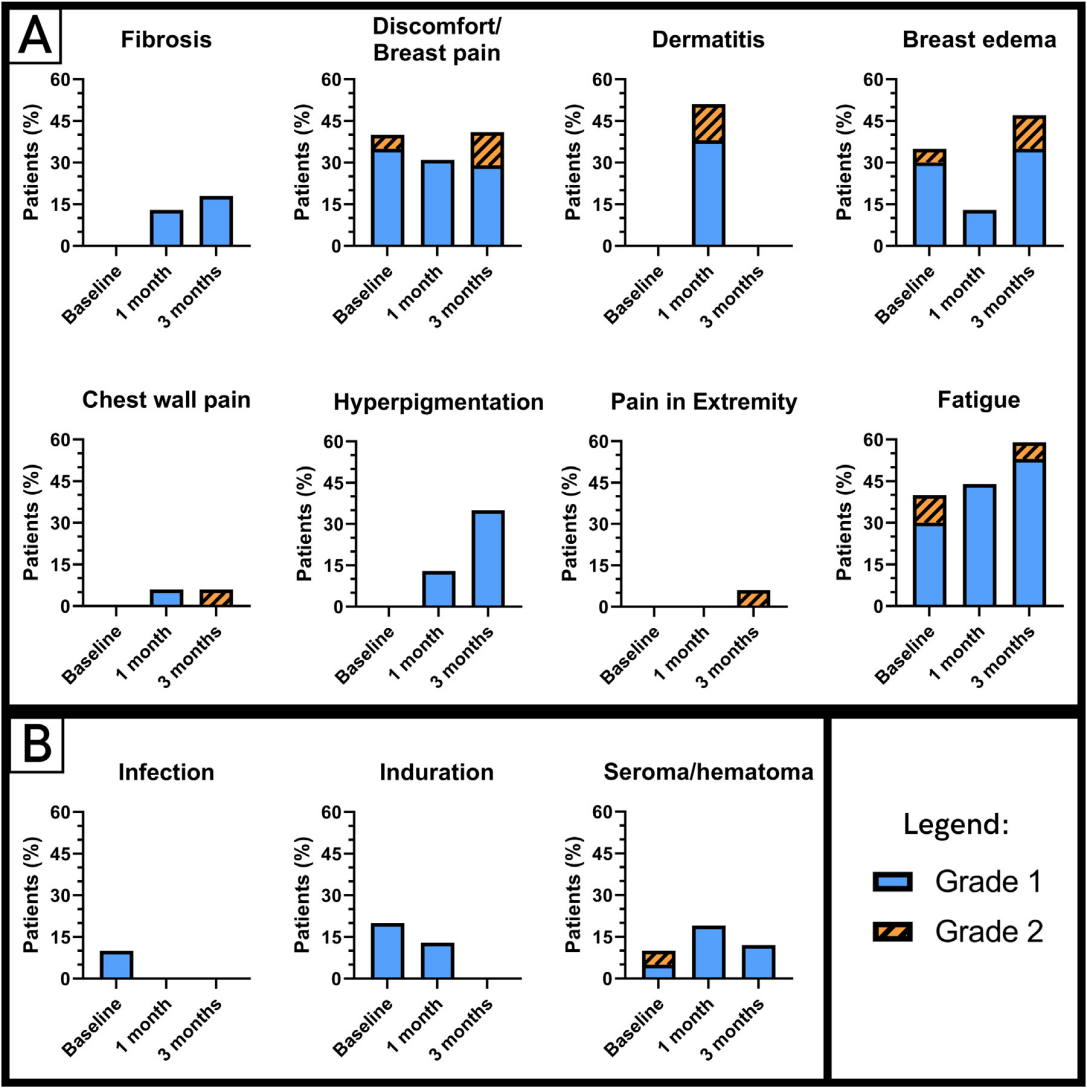
Toxicity according to the National Cancer Institute Common Terminology Criteria for Adverse Event v5.0 at baseline (ie, before radiation therapy and after surgery) and 1 and 3 months after radiation therapy. Grade 1 toxicity is shown in blue/even colored bars; grade 2 toxicity is shown in orange/dashed bars. (A) Radiation therapy-related toxicity and (B) surgery-associated toxicity.

The observed treatment-associated toxicity is shown in Fig. 4, consisting of grade 1 to 2 toxicity; no grade 3 or higher toxicity was observed. Three patients required a longer follow up because of persistent grade 1 breast edema, fibrosis, breast pain (n = 2), chest wall pain, grade 2 edema, and pain in the extremity (n = 1), which was probably caused by autoimmune disease. This toxicity was reversible within 3 to 6 months after RT.

## Discussion

This study showed the clinical feasibility of CBCT-based oART in 20 right-sided breast cancer patients treated with postoperative whole breast irradiation. Data collected on treatment time, treatment plan quality, toxicity, and patient satisfaction supported the feasibility and safety of the proposed workflow. To our knowledge, these results show the first prospective results of oART in breast patients, including a clinical evaluation of contour and plan quality in breast oART. The median treatment time recorded was 20.0 minutes, and the median on-couch time was 13.8 minutes. All adaptive treatment plans met our clinical criteria. Toxicity was mainly grade 0 and 1, and patient satisfaction was good.

An in silico study on breast oART showed similar times for target propagation and dose calculation.^11^ Treatment plan optimization was twice as long in this study compared with our results, which can be attributed to a longer optimization time for Volumetric Modulated Arc Therapy (VMAT) compared with the Intensity Modulated Radiation Therapy (IMRT) technique in our study.^11^ The oART workflow of the 20 breast cancer patients in our study was, on average, 7 minutes faster compared with rectum oART, which can be attributed to fewer changes in the targets and longer plan optimization due to the use of VMAT.^21^ In addition, we achieved slightly lower (1–3 minutes) treatment times compared with pelvic oART studies in prostate, bladder, and cervical cancer.^18^^,^^22^, ^23^, ^24^ Pelvic oART workflows do employ artificial intelligence-driven contouring during the online workflow and have higher mobility of the targets. The breast oART workflow was also faster compared with a palliative oART workflow in patients with painful metastases using diagnostic imaging and comfortable positioning without skin marks.^16^^,^^25^ Training of RTT staff for a breast oART RTT-only workflow was found to be feasible, which allowed the RTTs to conduct the most frequent manual contour corrections without supervision, which is currently performed at our department.

In our study, fractions with increased contouring time were mainly because of a learning curve for RTTs supervised by breast radiation oncologists combined with editing small differences between the automatic and manually corrected contours. The median treatment time recorded was still longer compared with our normal IGRT timeslots of 15 minutes for breast cancer patients. Regular timeslots could be feasible in the future if time spent on manual corrections of contours is reduced. The omission of the influencer structure had a positive effect on the on-couch time. The median time was 11 minutes (range, 11–14) without and 14 minutes (range, 11–25) with the influencer structure. Consequently, the omission of the influencer structures resulted in a further reduction of treatment time.

The oART workflow is inherently different from an IGRT workflow in breast cancer patients; less effort is required for positioning, and patients can be treated without skin marks for positioning. Our historical data analysis showed that for WBI, on average, 9% of patients require a new planning CT and treatment plan during treatment. Unfortunately, additional data on the frequency of repositioning patients in the IGRT workflow, followed by a repeat CBCT, were not available. Overall, the TP_A_ showed a significant improvement in PTV goals and no significant change in OAR dose compared with the scheduled plans. We were unable to determine which of the 20 patients, if treated with the IGRT workflow, would have needed a new planning CT because of contour changes. This was not possible since initial positioning in the oART workflow was not required to fully reproduce the reference positioning. Therefore, the disparities observed between TP_S_ and TP_A_ should be carefully considered and are partially because of differences in positioning. OART enables the possibility of omitting skin marks for positioning, which could be a psychological advantage for the patient.^26^ However, the introduction of SGRT could also enable this with the added value of motion management during treatment.^3^^,^^4^^,^^23^ In contrast to SGRT, the adaptive route provides the advantage of online adaptation when target shape changes occur. Omission of skin marks is currently standard practice for breast cancer patients treated with WBI using oART in our department.

Our results also showed that most of the manual contour corrections had a minimal impact on the optimized adapted plan, as evaluated on target coverage and OAR sparing. Manual and automated corrected contours showed differences in cranial and caudal slices of the targets, which could be caused by rotation of the target area. Reducing edits in the 2 outer caudal and cranial slices, as well as the omission of influencers and skin marks, resulted in a quicker workflow. Future challenges will be selecting the patients who are prone to target changes for oART and decreasing the PTV margin in patients without compromising the intrafraction motion.^27^ In our study, no patients required an additional planning CT during treatment. A larger benefit can be expected for the whole breast, including axillary nodes, where the current occurrence of replanning is 15% in our department.

Initially, intrafraction breast movement between CBCT2 and the end of radiation delivery was a concern, where the lack of a skin flash technique allowed only a small margin of error. Our data showed intrafraction movement in each direction with a standard deviation of <2 mm. This motion can consist of both patient motion and breast motion because of respiration; the latter is usually 1 to 5 mm.^28^, ^29^, ^30^ We also observed relaxation of the patient during treatment, which is also a reason for the reported vertical intrafraction motion of 0.13 cm. The development and implementation of a practical mini-bolus, with no density and not influencing the planned dose to the breast, unlike a virtual “full” bolus (Methods E3), enabled the opening of leaves past the breast contour and was able to reduce these risks with a margin of error of 3 mm.

The observed toxicity in patients treated with oART was mild and comparable with treatment-related toxicity reported in standard postoperative whole breast irradiation.^31^, ^32^, ^33^, ^34^ No grade 3 or higher toxicity was observed. Although oART takes longer compared with IGRT, this was not reflected by the dissatisfaction reported in the questionnaires. We expect that an increased experience of the multidisciplinary oART team, including improvement in manually correcting the targets by RTTs, will decrease on-couch duration.

Limitations of this study include the TF in which the patient enters and leaves the treatment room, registered by the RTTs. During a busy clinic and while learning a new workflow, these times were sometimes estimated, making a comparison with IGRT workflows difficult. A more accurate reflection of the oART treatment time was derived directly from the CBCT DICOM info header.

Future work will focus on the implementation of oART in other breast targets, including axillary levels 1 to 4, left-sided WBI using breath-hold technique, partial breast irradiation, and simultaneously integrated boost treatment schedules. Besides the implementation of the RTT-only workflow for right-sided WBI, the introduction of these new indications will also aim to result in RTT-only workflows fitting within regular treatment timeslots with the radiation oncologist and medical physics expert on call instead of at the machine. In addition, the current study was performed with standard CBCT imaging; recent research showed promising results in image quality when using a new high-performance ring-gantry CBCT. The impact of this technical and software-based improvement on the workflow should be investigated in the future.^35^^,^^36^ Additional potential benefits of oART, including a potential reduction of PTV margins, should be further investigated to reduce OAR dose, which may further reduce RT-associated toxicity.^37^^,^^38^

## Conclusions

An oART workflow was implemented for right-sided whole breast irradiation. The median on-couch time was acceptable. We have identified challenges and presented solutions for this workflow regarding robustness using skin flash, unnecessary manual contour corrections, and the omission of influencer structures.

## Disclosures

This research was funded by Varian, a Siemens Healthineers Company. Wilko F.A.R. Verbakel has received honoraria/travel expenses from Varian that are not related to the current work. Wilko F.A.R. Verbakel has been employed by both Varian and the Amsterdam UMC since May 2023.

## Appendix Supplementary materials

20241101 SUPPL_FOR_TYPESETTING_V2.pdf Image, application 1

## Footnotes

Supplementary material associated with this article can be found in the online version at doi:10.1016/j.adro.2024.101664.
