# Supplementary material for: Clinical Implementation of Cone Beam Computed Tomography-Guided Online Adaptive Radiation Therapy in Whole Breast Irradiation
Source: Adv Radiat Oncol. 2024 Nov 5;10(1):101664. doi: 10.1016/j.adro.2024.101664 (PMC11647482; doi:10.1016/j.adro.2024.101664)
Supplement: 20241101 SUPPL_FOR_TYPESETTING_V2.pdf [file mmc1.docx]

**Methods E1: Internal historical data re-planning in breast radiotherapy**

The institutional number of offline re-planning for all breast cancer patients during radiotherapy treatment at the Amsterdam UMC, was retrospectively analyzed from January 2018 to September 2022. A search was performed in the clinical Aria database (v16.1, Varian) for patients treated with curative intent who had re-planned treatment plans, i.e. two finished/irradiated treatment plans connected to a single intent in the database. Additionally, the type of treatment was identified in both right- and left-sided breast cancer patients treated with local (i.e. whole breast irradiation (WBI), post-mastectomy radiotherapy (PMRT), partial breast irradiation (PBI), or locoregional (i.e. axillary and internal mammary lymph nodes).

Analysis of historical treatment data between January 2018 and August 2022 yielded 2,304 courses of 2,192 breast cancer patients. This included 1,590 courses of local radiotherapy (WBI/PMRT), 700 courses of locoregional radiotherapy, and 14 courses which were excluded due to missing data. Re-planning occurred in 145 local radiotherapy (9%) cases and in 106 locoregional (15%) cases (left- or right-sided breast cancer). PTV margin was 5 mm for breast/chest wall, and 8 mm for regional lymph nodes.

**Methods E2: Beam setup script**

The specific beam angles per patient were optimized by an in-house developed script (Figure B1). This script is routinely used in our department's standard IGRT WBI treatment planning on Ethos. This script was based on earlier work and written in C# and linked to Eclipse via the Autoplan module (Varian, a Siemens Healthineers Company). This script has similar functionality compared to our Hybrid IMRT script^1^. The following steps are performed by the script:

1. The starting point for the first (medio-lateral) beam was a search between 30 and 60 degrees. The opposing beam latero-medial beam was always positioned opposite to the chosen angle with an additional 5 degrees added to avoid exact opposing beams.
2. The isocenter of the beams was chosen such that the focus to skin distance was the same for two opposing beams and the isocenter was approximately 7 mm from the Lung contour
3. The Contra-lateral breast + 3 mm (to account for breathing), heart, ipsilateral lung and liver were contoured as OARs.
4. A cost function was used to find the angle for which the OAR dose was minimal whilst still maintaining PTV coverage. This was done by optimizing the dose for each chosen beam angle using the optimization goal of PTV D98% >= 95%
5. Two additional beams were added 15° from the ML and LM beams.
6. The resulting beams were exported from Eclipse into the Ethos treatment planning system, here optimization of the dose based on a clinical goals template was done.

*[1] van Duren-Koopman MJ, Tol JP, Dahele M, Bucko E, Meijnen P, Slotman BJ, Verbakel WF. Personalized automated treatment planning for breast plus locoregional lymph nodes using Hybrid RapidArc. Practical radiation oncology. 2018 Sep 1;8(5):332-41.*


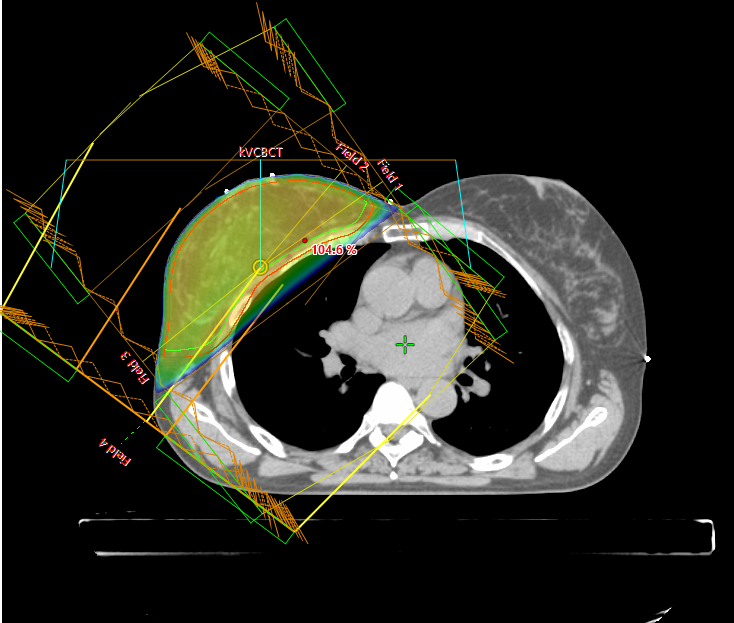


Figure E1: Scripted four beam IMRT tangential setup used for treatmentplanning. Dose color wash shown for 60%-110% dose, red contour is PTV cropped 5 mm from the body, green contour CTV cropped 5 mm from the body.

**Method E3:** **Mini-bolus**

The mini-bolus is a structure with no assigned density added during treatment planning which is delineated by an in-house developed script. The structure is first contoured in the axial slice at the beam isocenter. The script draws a 7-voxel wide rectangular contour from the isocenter to 55 mm outside the body, direction of this contour is perpendicular to one of the tangential beam directions (Supplementary materials, Appendix B). Next, this contour is added to every axial slice till 4 slices cranially and caudally of the target. Finally, the structure is added to the body contour. The additional outer body contour forces the optimizer to open the MLCs further, which allows for some intra-fraction variation up to 3 mm in a ventrolateral direction.


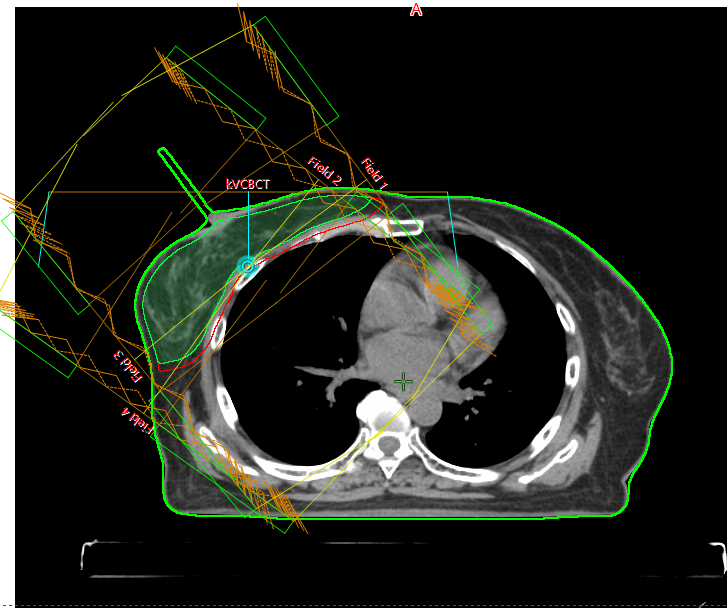


Figure E2: Example of the mini-bolus added to the body contour


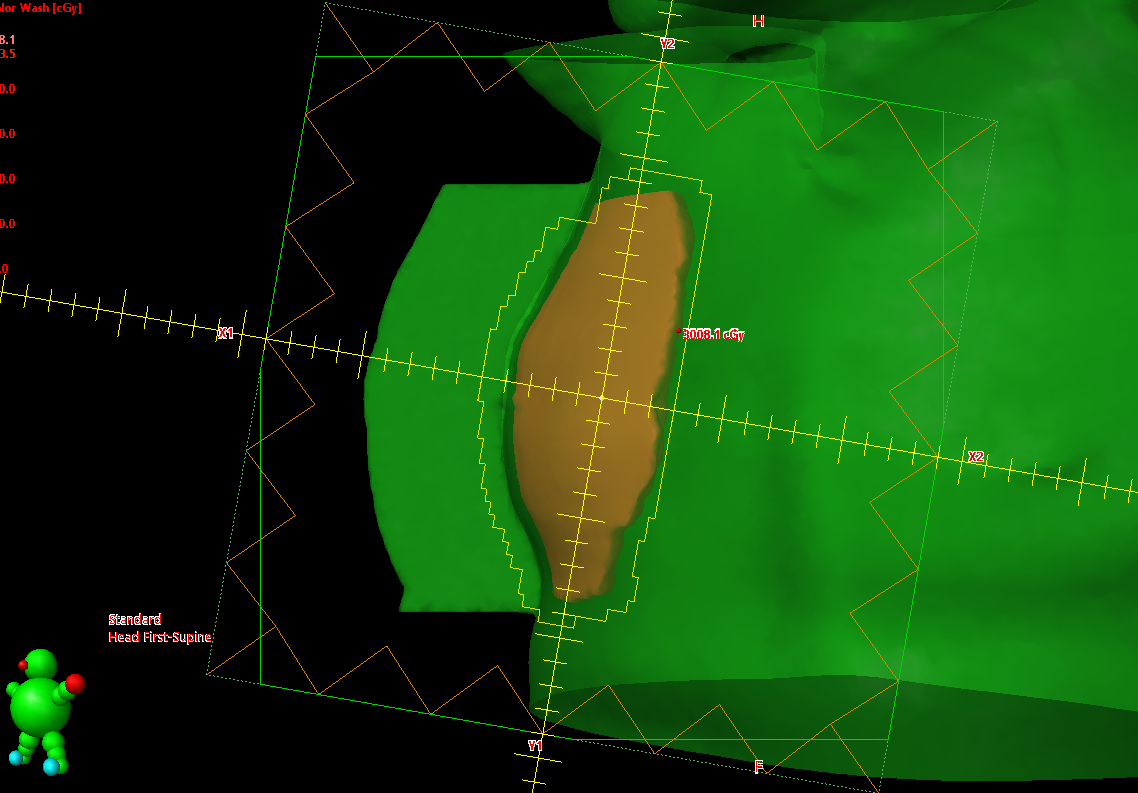


Figure E3: Anterior beam eye view with example of opening of MLCs beyond original body due to mini-bolus. Green is showing a 3D render of the body including the mini-bolus addition, red contour is the PTV.


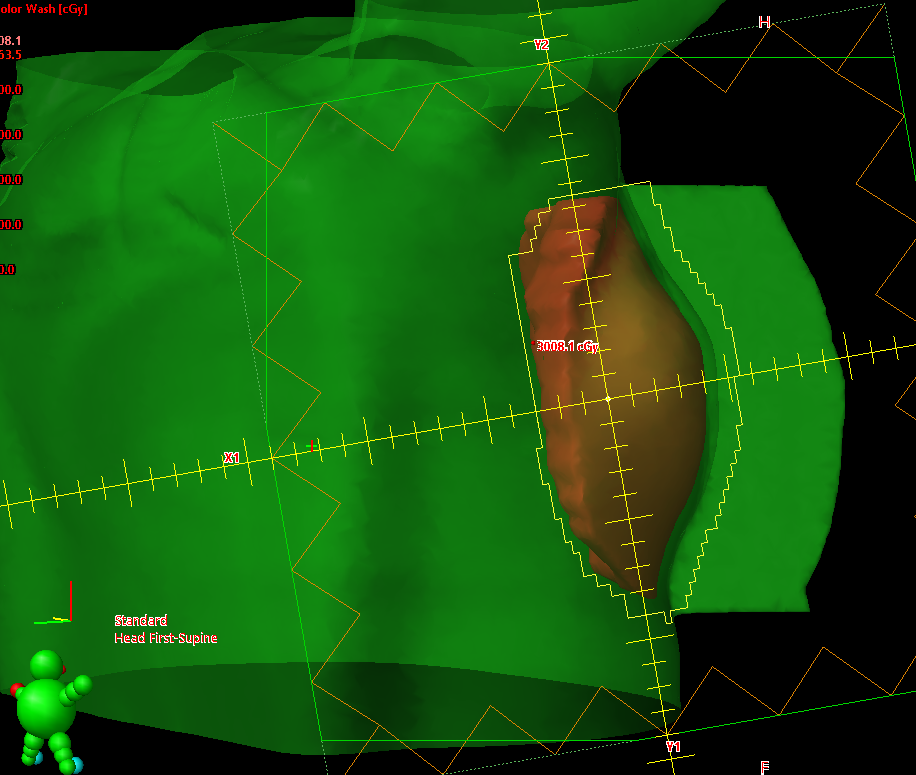


Figure E4: Posterior beam eye view with example of opening of MLCs beyond original body due to mini-bolus. Green is showing a 3D render of the body body including the mini-bolus addition, red contour is the PTV.

**Methods E4: Questionnaire questions and results**

In-house developed questionnaire (translated from Dutch). All questions had the following answer options: 1 – not at all, 2 – a little, 3- quite, 4 – a lot. Negative outcomes or dissatisfaction was defined by scoring 1 for questions 1-3, 5-7, and 10; and for questions 4, 8, and 9 by scoring 4.

Translated questionnaire (developed in-house)

Given the intensity of treatment on this radiation device, we would like to evaluate your experience. Could you please indicate how much each of these questions applies to your experience?

1. Was the explanation of the treatment preparation and procedure understandable?
2. Did the radiation oncologist adequately inform you regarding possible side effects?
3. Were you satisfied with the length of time between your consultation with the radiation oncologist and the commencement of your treatment?
4. Did you experience anxiety during your time on the treatment table?
5. Was the amount of time spent on the treatment table acceptable?
6. Was communication during treatment understandable?
7. Did you think the radiation device's support staff was professional in their approach?
8. Did symptoms from your disease increase during the treatment?
9. Did you find the total time spent at the department to be long?
10. In case you are referred for radiotherapy again, would you prefer the same treatment pathway?

Original questionnaire in Dutch (Version 2, June 16, 2022):

Vanwege de intensieve behandeling op dit bestralingsapparaat, willen wij graag evalueren hoe u dit hebt ervaren. Wilt u aangeven in welke mate onderstaande vragen op u van toepassing zijn?

Options: 1 – helemaal niet, 2- een beetje, 3 – nogal, 4 - heel erg

1. Was de uitleg over de procedure van de voorbereiding en de behandeling begrijpelijk?
2. Was u door de bestralingsarts goed voorbereid op de te verwachten bijwerkingen?
3. Was de wachttijd tussen het eerste consult bij de bestralingsarts en start van de behandeling acceptabel?
4. Voelde u zich angstig tijdens het liggen in het bestralingstoestel?
5. Was de tijd dat u op het bestralingstoestel lag acceptabel?
6. Was de communicatie tijdens de behandeling begrijpelijk?
7. Heeft u de begeleiding van het personeel op het bestralingstoestel als professioneel ervaren?
8. Zijn de klachten die u als gevolg van uw ziekte heeft, toegenomen tijdens de behandeling?
9. Heeft u de totale duur dat u op de afdeling aanwezig was als lang ervaren?
10. Indien u opnieuw verwezen wordt voor een bestraling, vind u het dan prettig om op dezelfde manier behandeld te worden?


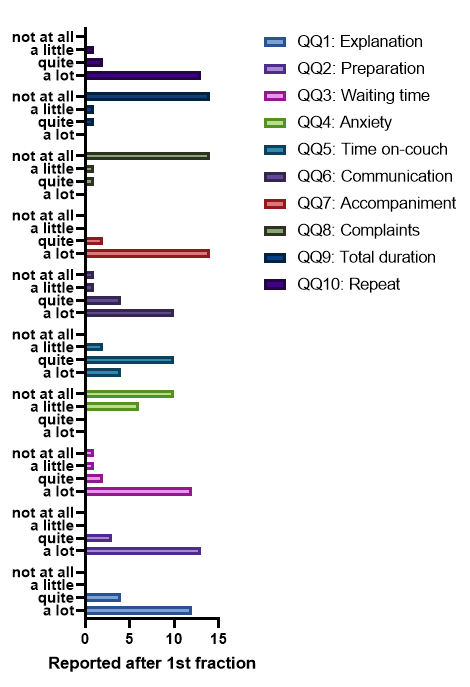


Figure E5: Questionnaire results after the first fraction in 16 patients.


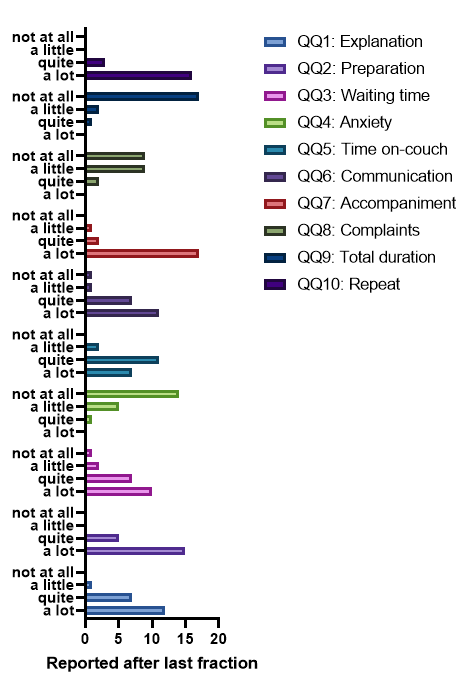


Figure E6: Questionnaire results after the last fraction in 20 patients.
